# Electrochemically Formed Porous Silica

**DOI:** 10.3390/ma4050825

**Published:** 2011-04-26

**Authors:** Jean-Noël Chazalviel, François Ozanam

**Affiliations:** Physique de la Matière Condensée, Ecole Polytechnique, CNRS, 91128 Palaiseau, France; E-Mail: francois.ozanam@polytechnique.fr

**Keywords:** silicon, anodic oxide, electrochemical dissolution, morphologies

## Abstract

Controlled electrochemical formation of porous silica can be realized in dilute aqueous, neutral-pH, fluoride medium. Formation of a porous film is initiated by sweeping the potential applied to silicon to values higher than 20 V. Film formation, reaching a steady state, may be pursued in a wide range of potentials, including lower potentials. The origin of a threshold potential for porous film initiation has been explained quantitatively. All of the films appear mesoporous. Films grown at high potentials exhibit a variety of macrostructures superimposed on the mesoporosity. These macrostructures result from selective dissolution of silica induced by local pH lowering due to oxygen evolution. Films grown at potentials lower than 15 V appear uniform on the micrometer scale. However, all of the films also exhibit a stratified structure on the scale of a few tens of nanometres. This periodic structure can be traced back to the oscillatory behavior observed during the electrochemical dissolution of silicon in fluoride medium. It suggests that periodic breaking of the growing film may be responsible for this morphology.

## 1. Introduction

Anodic films formed at high potential are prone to exhibiting porosity. Porous alumina formed by anodization of aluminium has long been known and widely used in industrial applications [1,2,3]. In recent years, there has been renewed interest in the porous oxide films formed on aluminium [4,5], titanium [6,7], and other “valve metals” [8,9,10,11,12], when it was realized that these porous structures can be ordered and that nanotubes may be formed as well as pores. The case of silicon had been somewhat put aside, because porous silica is easily prepared by other routes, e.g., sol-gel processes [13], and there was no obvious interest in preparing silica from silicon. Noticeably, for electronic applications, compact oxides are usually desirable. Furthermore, electrochemical oxides received some bad press due to the possible presence of fixed charges induced by incorporation of ions [14], and only in very special cases were electronic-quality oxides achieved by anodic treatment [15]. However, renewed interest in porous silicon [16], together with some intriguing features of silicon electrochemistry in fluoride electrolytes, have led us and others to explore the electrochemical behavior of silicon in these media [17,18,19,20,21,22,23,24]. Though silicon oxide is soluble in fluoride medium, an oxide film may appear at the silicon surface under anodic polarization [25], and under suitable conditions it turns out that this film may be porous [26,27]. An advantage of using a fluoride medium is due to the fact that silica ultimately dissolves, which leads to a steady state of the oxide film. This makes the final state insensitive to the initial preparation of the surface, thereby providing a degree of reproducibility almost unattainable in non-fluoride electrolytes. We will review here the work performed on this subject over the last few years.

## 2. Anodic Behavior of Silicon in Acidic Fluoride Electrolyte

**Figure 1:**
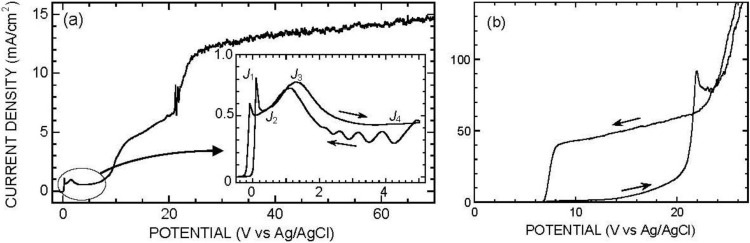
(**a**) Voltammogram of p-Si in acidic fluoride medium 1 M NH_4_Cl + 0.025 M NH_4_F + 0.025 M HF (*c*_F_ = 0.05 M, pH 3). RDE 300 rpm, scan rate 20 mV/s. The inset shows a magnified voltammogram restricted to the low-potential range, with the characteristic currents *J*_1_, *J*_2_, *J*_3_, *J*_4_. Note the two extra current increases around 10 V and 20 V; (**b**) Voltammogram of the same material in neutral fluoride medium 0.25 M NH_4_F + 0.01 M NH_4_OH (pH ≈ 8). RDE 200 rpm, scan rate 100 mV/s. Note the strong hysteresis between 7 V and 25 V.

The voltammogram of silicon in acidic fluoride electrolyte has been described by many authors [17,18,19,20,21,22] and is represented in Figure 1a. As can be seen in the inset, it exhibits a fast-current-rising region followed by two electropolishing plateaus. The fast-current-rising region, located in a narrow potential range around 0 V *vs*. Ag/AgCl, is the region of porous silicon formation. In this region, the surface is essentially covered with SiH bonds [28,29], and the surface intermediates corresponding to silicon oxidation (SiF or SiOH groups) are short-lived. Upon increasing the potential, a sharp current maximum is observed (current density *J*_1_), and a first current plateau (*J*_2_, extending typically from 0 to 1 V *vs*. SCE) appears. In this region, a hydrated (“wet”) oxide is present at the surface [25]. Upon a further potential increase, a broad current maximum is observed (*J*_3_), and a second current plateau (*J*_4_) is reached. It seems that this transition corresponds to the appearance of a dry oxide film underneath the wet one [25]. In this range, the electrode is electropolished and the overall oxide thickness is on the order of 1 nm/V. A puzzling feature of this second plateau is the observation of a tendency to an oscillatory behavior of the electrochemical system (see backwards potential scan in the inset of Figure 1a); a behavior which has attracted the interest of many researchers [19,30,31,32,33,34,35,36,37,38,39,40]. In brief, it appears that the oxidation/dissolution process is self-oscillatory on a local scale, and can be synchronized over the electrode surface by an external excitation or by global coupling, e.g., through a series resistance [31], but the very origin of the self-oscillatory process (electric or stress-induced breakdown?) is still a controversial point. All of these features (from *J*_1_ to *J*_4_) are observed in any fluoride electrolyte, for p- and n-type silicon. However, for n-Si, they can be observed under illumination only, due to the need of holes to initiate silicon oxidation (holes may also be supplied by avalanche breakdown at high potential or tunneling in n^+^-Si, a feature which has been used in porous silicon preparation, though we are not aware of such studies in dilute fluoride medium). Another important point is the variation in the magnitude of the characteristic currents *J_i_*, which vary with fluoride concentration *c*_F_ (typically as *c*_F_^α^, with α~1 to 2.5), pH (fast fall off above pH 3–4), and electrode rotation rate (mixed control, associated with a Koutecky-Levich behavior [41,42]). In practice, the characteristic currents become too high to be observed in concentrated HF solution (typical order of magnitude 0.1–1 A/cm^2^ for 10% HF solution).

The above summarizes the most thoroughly studied part of the voltammogram, which extends up to ca +5 V *vs*. SCE. However, non-trivial features appear above this “classical” range. Above a critical potential on the order of 10 V *vs*. SCE, the current exhibits a second sharp rise, and a new plateau is reached (Figure 1a) [43]. A combined *in-situ* infrared/electrochemical impedance study has revealed that this current rise is associated with a strong increase in the amount of oxide, though the electrical thickness of the oxide film (as deduced from the high-frequency interface capacitance) does not increase that much [26]. These two pieces of information indicate that a thick porous oxide film has appeared above the initial compact film. Typical orders of magnitude are 10 nm for the compact film and 100 nm for the porous film. Also, the electrochemical impedance study has shown that the tendency to an oscillatory behavior observed in the second electropolishing plateau region survives in this regime, as a broadened resonance in the low-frequency range [26].

Finally, above 20–25 V, a new increase in current appears (Figure 1a), associated with strong gas evolution at the electrode. This obviously corresponds to the anodic decomposition of water, associated with oxygen evolution [43].

Interestingly, electroluminescence has been observed in these two high-potential regimes: a red luminescence, attributed to silicon-rich zones in the oxide, and a blue luminescence, attributed to oxide defects. The change in electrochemical regime above 20–30 V is associated with a change in luminescence from a dominant red contribution to a dominant blue contribution [43].

However, the oxide films produced in such conditions can hardly be removed from the electrolyte, because they are very thin and are partially dissolved before the electrode is removed from the electrolyte and the surface is rinsed. This has motivated studies in neutral fluoride electrolytes.

## 3. Anodic Behavior of Silicon in Neutral Fluoride Electrolyte

The solubility and dissolution rate of silica in fluoride medium become very low at neutral pH. As a result, the characteristic currents *J_i_* associated with the classical potential range are extremely small, and the electrode is essentially passivated [22]. Figure 1b shows a typical voltammogram obtained in 0.25 M NH_4_F, buffered to pH ≈ 8 by addition of 0.01M NH_4_OH. Interestingly, when the potential is swept above a critical value, on the order of +20 V, the current abruptly increases and a new regime is entered: the potential can then be swept back and forth through a wide range, and another current/potential relation is obeyed [27,44]. The passive regime is recovered only upon sweeping the potential down to a much lower value (typically +7 V for 0.25 M NH_4_F electrolyte). This behavior can be understood if one realizes that the dissolution of silicon is actually a two-step process:

Si + 4h^+^ + 2H_2_O → SiO_2_ + 4H^+^ (electrochemical oxidation of silicon) (1)

SiO_2_ + 6F^−^ +4H^+^ → SiF_6_^2−^ + 2H_2_O (chemical dissolution of the oxide) (2)

**Figure 2:**
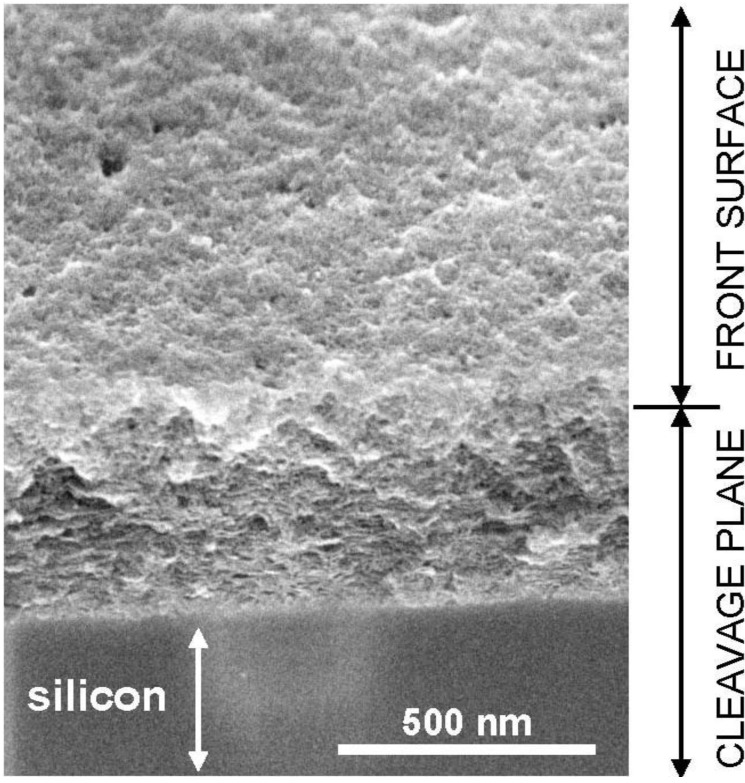
Microstructure of a typical porous silica film (*c*_F_ = 0.25 M, 12 V, 1,600 rpm), obtained with a field-emission SEM (view at 45° near a cleavage).

Once a porous structure has been initiated, the electrochemical reaction takes place near the electrode, that is, at the bottom of the pores, whereas the chemical reaction can proceed anywhere in the depth of the layer. Since the medium is neutral, the protons available for reaction (2) to take place are just those created by reaction (1). If part of them escape to the electrolyte, the rate of oxide dissolution will be limited by proton supply, and will become lower than the rate of oxide formation, leading to growth of the porous layer. In practice, if the potential is swept back to low values or if the potentiostatic control is turned off, the pH returns to its initial (homogeneous) neutral value and the passive state is recovered. Interestingly, the oxide formed is preserved, and the electrode can be removed from the electrolyte and rinsed without damaging it. SEM observations indicated that a porous structure is indeed present (Figure 2), but the characteristic scale of the porosity is too small to be resolved, which means a scale in the nanometric range [27]. Note that, since a steady state is reached, the duration spent at the formation potential is not a critical parameter of the experiment: perfectly identical oxide films are obtained for any duration exceeding a few tens of seconds.

An obvious question is about the pore initiation mechanism. At this stage, a reasonable hypothesis was that a local acidification of the medium is produced by some oxygen evolution taking place:

4h^+^ + 2H_2_O → O_2_ + 4H^+^ (3)

This reaction is expected to occur in this potential range (see Figure 1a), and local acidification can lead to local dissolution of SiO_2_ [27].

## 4. Porous Silica: Formation Mechanism

In order to assess these mechanisms, the above qualitative arguments have been turned into a mathematical formulation [45] that will be presented here, since it has not yet been published in detail. The silicon surface and oxide films were assumed to be planar, and the thicknesses of the compact oxide and porous oxide were taken as *d*_0_ and *d*, respectively. The concentration profiles of the various ionic species as a function of distance *x* from the silicon surface were investigated. Electrolyte stirring was assumed to lead to a diffusion layer of thickness δ (diffusion-layer approximation, *i.e*., zero convection inside the layer, concentrations uniform outside the layer).

### 4.1. Case of Acidic Fluoride Electrolytes

As a first step, the case of acidic fluoride electrolytes was considered. It was demonstrated that, though fluoride concentration is depleted at the bottom of the pores, the medium is not fully ion-depleted (NH_4_^+^, SiF_6_^2−^ and H^+^ ions are still present), so that neglecting the electric-field migration terms in the electrolyte is a reasonable approximation. In the steady state, the fluoride concentration (here we mean the sum of HF, F^−^, HF_2_^−^ concentrations, assuming a common diffusion constant *D*_F_ for all of these species) in the porous oxide *F*(*x*) (*d*_0_ < *x* < *d*_0_ + *d*) is then ruled by a diffusion-reaction equation (second Fick’s law):

(4) $$D_{F}\frac{d^{2}F(x)}{dx^{2}}=6W_{\text{diss}}(x)$$

where *W*_diss_ is the dissolution rate of porous silica (number of SiO_2_ units dissolved per unit time and per unit *volume*, a function of the local electrolyte composition). The fluoride concentration outside the oxide (*d*_0_ + *d* < *x* < *d*_0_ + *d* + δ) is ruled by the same equation, with no second member (free diffusion), and a boundary condition *F* = *c*_F_ (fluoride concentration in the bulk electrolyte) at the outer boundary *x* = *d*_0_ + *d* + δ. The inner boundary condition at *x* = *d*_0_ can be written from first Fick’s law

(5) $$D_{F}{\frac{dF}{dx}|}_{d_{0}}=6W_{d}$$

where *W*_d_ is the dissolution rate of compact silica (number of SiO_2_ units dissolved per unit time and per unit *surface area*). Note that one must have *W*_diss_/*W*_d_ ≈ *a*, the specific surface area of the porous oxide (units of length^−1^). In the steady state (oxide formation balancing oxide dissolution), the Faradaic current flowing through the interface corresponds to the sum of the dissolution at the compact oxide surface and inside the porous oxide:

(6) $$J=4e\left(W_{d}+\int_{d_{0}}^{d_{0}+d}W_{\text{diss}}(x)dx\right)=\frac{2eD_{F}}{3}{\frac{dF}{dx}|}_{d_{0}+d}=\frac{2eD_{F}}{3}{\frac{dF}{dx}|}_{d_{0}+d+\text{δ}}$$

Two extra conditions must be expressed at the electrochemical interface:

*J* = *J*_0_ [exp(*E*/*E*_0_) − 1] (7)

*J* = 4*eW*_d_ [1 + exp((*V−V*_c_)/*E*_0_*d*_c_)] (8)

Equation (7), where *E* is the electric field in the compact oxide layer and *J*_0_ and *E*_0_ are two characteristic constants of the material, is a classical expression for ionic transport through the oxide [46,47]. Equation (8), where *V = Ed*_0_ is applied potential, is a heuristic assumption, allowing for the smooth appearance of a porous oxide layer: In the absence of a porous layer, equating *J* to 4*eW*_d_ would lead to *E* = *E*_0_ ln(1 + 4*eW*_d_/*J*_0_) (constant), that is, an oxide thickness *d*_0_ = *V*/*E* simply proportional to applied potential. With Equation (8), this behavior is preserved as long as the potential is lower than *V*_c_. However, above the critical potential *V*_c_ and an oxide thickness *d*_c_ [taken for consistency as *V*_c_/ln(1 + 4*eW*_d_/*J*_0_)], the current exceeds the dissolution current at a flat SiO_2_ surface, the extra current being associated with the dissolution of the porous layer. Note that this expression describes the appearance of a porous layer but the physical origin of this appearance, a highly controversial point in the formation of porous anodic oxide films, is left open [48,49,50].

**Figure 3:**
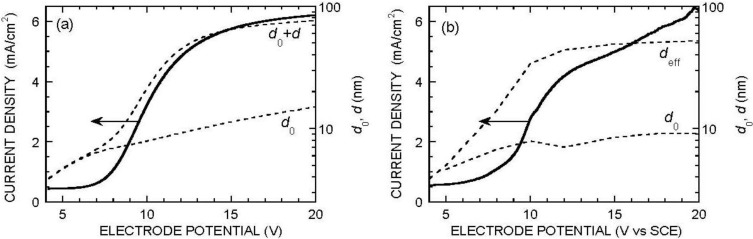
(**a**) Simulated voltammogram and oxide thicknesses (*d*_0_ compact layer, *d* porous layer) in acidic fluoride medium. *c*_F_ = 0.05 M, pH = 3, δ = 75 μm, *E*_0_ = 10^6^ V/cm, *J*_0_ = 10^−5^ mA/cm^2^, *V*_c_ = 7.5 V, *D*_F_ = 1.5 × 10^−5^ cm^2^/s, *W*_d_ = *KF*^2^ with *K* = 0.83 × 10^−24^ cm^4^/s (whence *W*_d_ = 7.5 × 10^14^ cm^−2^/s for *F* = *c*_F_), *W*_diss_ = *a*(*KF*^2^ + *K*_0_) with *a* = 10^7^ cm^−1^ and *K*_0_ = 1.5 × 10^14^ cm^−2^/s; (**b**) Experimental data plotted with identical scales. *d*_eff_ is the “effective” oxide thickness as determined by quantitative infrared spectroscopy.

Equation (4), with the boundary conditions (5–8), was solved by numerical integration from *x* = *d*_0_ + *d* to *x* = *d*_0_. The current density was used as a parameter, and the initial condition at *x* = *d*_0_ + *d* was taken from Equation (6) as d*F*/*dx* = 3*J*/2*eD*_F_, *F* = *c*_F_ − 3*J*δ/2*eD*_F_. The inner bound of the porous oxide layer was reached when condition (5) was met, which determined the actual value of *d*. The value of *V* was then extracted from Equation (8) and that of *d*_0_ was deduced by using *E* from Equation (7). Figure 3 shows a set of results reproducing the shape of the experimental data for *c*_F_ = 0.05 M, pH 3. Taking δ = 75 μm and *D*_F_ = 1.5 × 10^−5^ cm^2^/s, the values of the fitting parameters were found to be in the expected range, namely *W*_d_ = *KF*^2^, with *K* = 0.83 × 10^−24^ cm^4^/s (a fair representation of the experimental data in the electropolishing range [41,42]), *E*_0_ = 10^6^ V/cm, *J*_0_ = 10^−5^ mA/cm^2^, *V*_c_ = 7.5 V, *a* = 10^7^ cm^−1^ (= 1,000 m^2^/cm^3^). Note however that for reaching a reasonable fit, one had to assume a somewhat different law for *W*_diss_ and *W*_d_, namely *W*_diss_ = *a*(*KF*^2^ + *K*_0_), with *K*_0_ = 1.5 × 10^14^ cm^−2^/s. The existence of a non-nul dissolution rate at zero fluoride concentration is indeed in agreement with experimental data in the literature [42]. The fact that a good fit is obtained when this term is present in *W*_diss_ and absent from *W*_d_ suggests that silica dissolution is inhibited at the pore bottoms because the electrolyte is saturated with dissolved silica awaiting its conversion to fluorosilicate.

### 4.2. Case of Neutral Fluoride Electrolytes

As a next step, the above treatment was extended to the case of neutral electrolytes. In that case, one has to worry about the concentration profiles of the fluoride ions and the protons. Furthermore, the diffusion of these species is complicated by the fact that they may react together, according to the equilibrium.

HF ⇄ H^+^ + F^−^ (pK_a_ = 3.1). (9)

Even by neglecting the existence of the HF_2_^−^ species (an approximation acceptable for low fluoride concentrations), in the steady state, the free diffusion (*i.e*., outside the silica layer, with (9) as the only possible reaction) of the fluoride and proton species is ruled by a set of three equations:

*D*_F−_d^2^[F^−^]/d*x*^2^ = *w* (10)

*D*_H_d^2^*H*/d*x*^2^ = *w* (11)

*D*_HF_d^2^[HF]/d*x*^2^ = −*w* (12)

where again *H* stands for [H^+^] and *w* represents the forward rate of reaction (9) (generation rate for F^−^ and H^+^, consumption rate for HF). Eliminating *w* leads to a set of two diffusion equations. The quantities of interest appear to be the “averaged fluoride concentration” *F*_m_ = (*D*_F−_[F^−^] + *D*_HF_[HF])/(*D*_F−_ + *D*_HF_) and the “averaged proton concentration” (free or complexed) *H*_m_ = (*D*_H_*H* + *D*_HF_[HF])/(*D*_H_ + *D*_HF_). The free-diffusion equations then turn to d^2^*F*_m_/d*x*^2^ = 0 and d^2^*H*_m_/d*x*^2^ = 0. If we further assume *D*_F−_ ≈ *D*_HF_, we can keep the total fluoride concentration *F* (≈2*F*_m_) and the equations in the porous layer are then

*D*_F_d^2^*F*/d*x*^2^ = 6*W*_diss_

(*D*_H_ + *D*_F_)d^2^*H*_m_/d*x*^2^ = 4*W*_diss_ (13)

with the following boundary conditions at *x* = *d*_0_:

*D*_F_d*F*/d*x* = 6*W*_d_

(*D*_H_ + *D*_F_)d*H*_m_/d*x* = −*J*/*e* (14)

This set of equations provides a means to determine the concentration profiles, provided that one can calculate the proton concentration *H* from *F* and *H*_m_. This is done by assuming that reaction (9) is locally at equilibrium, whence:

*H* [F^−^] = *K*_a_ [HF] (15)

*F* = [F^−^] + [HF] (16)

*H*_m_ = (*D*_H_*H* + *D*_F_[HF])/(*D*_H_ + *D*_F_) (17)

Eliminating [F^−^] and [HF] allows one to calculate *H* as a function of *H*_m_ by resolving a second degree equation:

(18) $$H^{2}\frac{D_{H}}{D_{H}+D_{F}}+H\left(\frac{K_{a}D_{H}}{D_{H}+D_{F}}+\;\frac{D_{F}F}{D_{H}+D_{F}}-H_{\text{m}}\right)-K_{a}H_{m}=0$$

and deducing [F^−^] = *F*/(1 + *H*/*K*_a_) and [HF] = *F*/(1 + *K*_a_/*H*). In practice, the problem was solved numerically by following the same method as in the acidic case. In order to assess the mechanism of pore initiation, the presence of a small oxygen-evolution current *J*_O2_ was incorporated into the model, leading to the outer boundary conditions:

(19) $$F(d_{0}+d)\;=c_{F}-\frac{(J-J_{O2})\delta }{(2/3)eD_{F}}\text{ and }{\frac{dF}{dx}|}_{d_{0}+d}\;=\frac{\left(J-J_{O2}\right)}{(2/3)eD_{F}}$$

(20) $$H_{m}(d_{0}+d)\;=\frac{J_{O2}\delta }{e(D_{H}+D_{F})}\text{ and }{\frac{dH_{m}}{dx}|}_{d_{0}+d}=\frac{-J_{O2}}{e(D_{H}+D_{F})}$$

**Figure 4:**
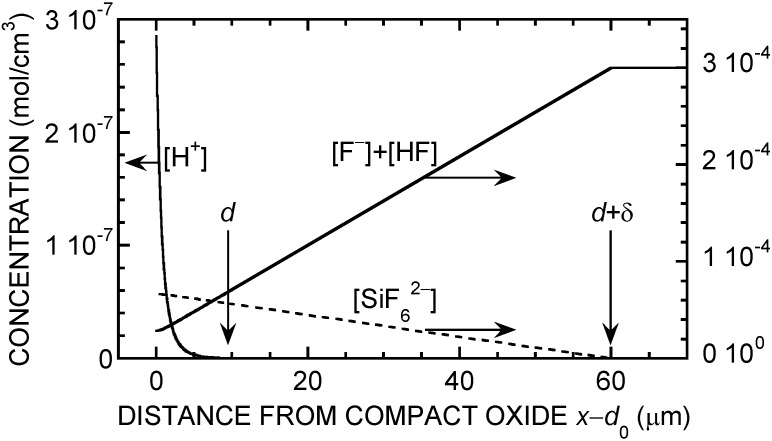
Typical simulated profiles for *F* and *H* when the porous oxide is formed in neutral fluoride electrolyte, as calculated for *c*_F_ = 0.3 M, *D*_HF_ = *D*_F_, *D*_H_ = 9.3 × 10^−5^ cm^2^/s, *J* = 45 mA/cm^2^, *J*_O2_ = 1 μA/cm^2^, δ = 50 μm, and other parameters as in Figure 3. The profile for the fluorosilicate ions (assuming a diffusion constant of 10^−5^ cm^2^/s) is also shown as a complementary piece of information. (These profiles correspond to 13.5 V, 45 mA/cm^2^ in Figure 5a).

**Figure 5:**
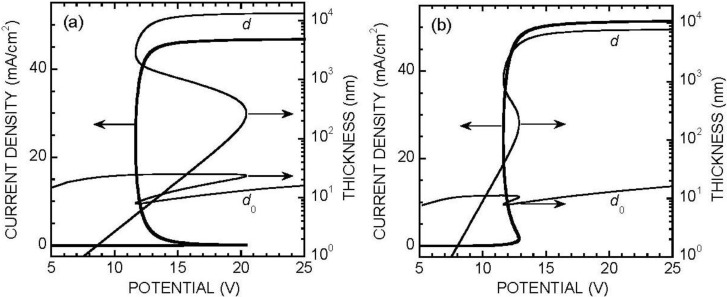
Typical simulated current density and oxide thicknesses (compact oxide *d*_0_ and porous oxide *d*) in the steady state, when the anodization is performed in neutral fluoride medium. *c*_F_ = 0.3 M, *D*_HF_ = *D*_F_, *D*_H_ = 9.3 × 10^−5^ cm^2^/s, δ = 50 μm, fixed value of the oxygen-evolution current *J*_O2_ = 1 μA/cm^2^ (**a**) and 10 μA/cm^2^ (**b**). Other parameters are as in Figure 3, except for *W* and *W*_diss_, now functions of pH, according to a law cst × 2*H*_c_^0.4^*H*^1.6^/(*H*^2^ + *H*_c_^2^), with *H*_c_ = 10^−6^ mol/cm^3^.

Numerical integration of Equations (4) and (13) was carried out by proceeding from the outer oxide boundary inwards. At each position, *H* was calculated from *H*_m_ and *F* by solving (18). The expressions for *W*_d_ and *W*_diss_ were extended to variable pH by incorporating a multiplying factor 2*H*_c_^0.4^*H*^1.6^/(*H*^2^ + *H*_c_^2^), with *H*_c_ = 10^−6^ mol/cm^3^ (10^−3^ M), a reasonable representation of the pH dependence of *J*_4_ [41,42]. The integration was stopped when condition (5) was met, determining the value of *d*. Figure 4 shows typical profiles for *F* and *H*. Note the linear variation of the concentrations in the diffusion layer and the weak curvature of the profiles (more pronounced near *x* = *d*_0_) within the porous oxide. Figure 5 shows typical results for two fixed values of *J*_O2_. The *J*(*V*) curve is seen to exhibit an S-shape. The middle part of the S is unstable, accounting for the experimentally observed hysteresis. The critical potential for switching from the lower branch to the upper branch of the curve is seen to depend critically on *J*_O2_, a confirmation of the origin of pore initiation.

**Figure 6:**
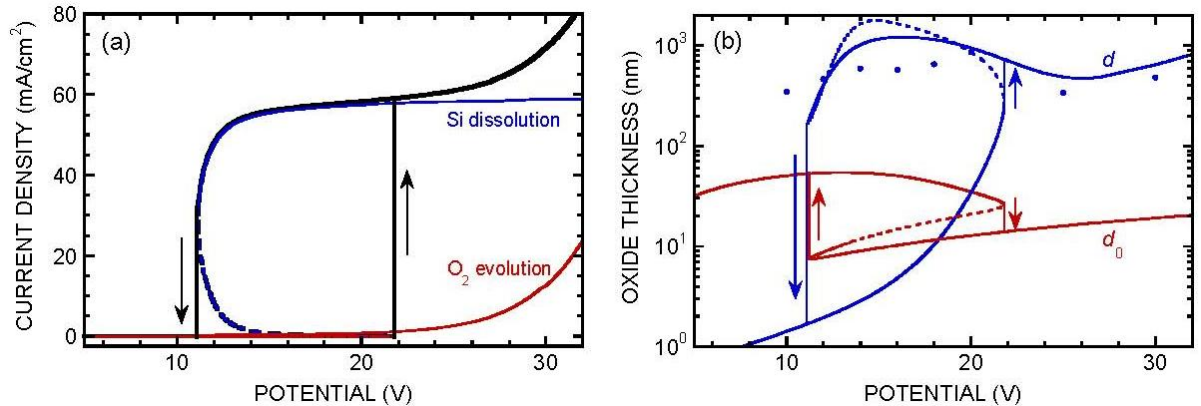
Simulated current density (**a**) and oxide thicknesses (**b**) (compact oxide *d*_0_ and porous oxide *d*) when the anodization is performed in neutral fluoride medium. Parameters as in Figure 5, except for *J*_O2_, now assumed to depend on potential as explained in the text. The dotted portions of the curves correspond to an unstable state and cannot be observed. In practice, the system is expected to jump between the passive regime and the dissolution regime at the potentials limiting this unstable region (see vertical lines and arrows). Note the qualitative similarity of (**a**) with Figure 1b. See also the experimental points for porous-oxide thickness added in (**b**).

In practice, *J*_O2_ is not a constant. In an attempt to better mimic the actual *J*(*V*) curve, we have assumed a law of the type *J*_O2_ = (*J*_c_ + *J*_FN_)[1 + exp((*V* − *V*_0_)/Δ*V*_0_)], where *J*_FN_ is a Fowler-Nordheim current, of the form *J*_FN_ = *AE*^2^exp(−*B*/*E*), *J*_c_ is a small constant current (leakage current at defects at electric-field values for which *J*_FN_ is negligible) and the multiplying factor represents impact ionization in the oxide when the potential reaches a critical value *V*_0_. Figure 6 shows the *J*(*V*), *d*_0_(*V*) and *d*(*V*) curves for values of the parameters *A* = 10^−5^ mA/V^2^, *B* = 3.5 × 10^8^ V/cm (typical values for Fowler-Nordheim injection from silicon into silica [51]), *J*_c_ = 3 × 10^−4^ mA/cm^2^, *V*_0_ = 20 V and Δ*V*_0_ = 3 V. Note the overall good agreement with experiment. Especially, and as expected from the law assumed for *J*_O2_, the ratio *J*_O2_/*J* is seen to increase when potential increases above 20 V. This is consistent with the experimental observation that, for potentials lower than 20 V, the current follows a Koutecky-Levich law, with a mass-transport contribution consistent with fluoride diffusion to the electrode, which is no more the case at higher potentials [52]. Interestingly, the porous oxide thickness is seen to go through a maximum on the order of a micrometer, decreases when oxygen evolution becomes appreciable, and reincreases at higher potentials, a behavior in agreement with experiment (see Figure 6b and reference [52]).

## 5. Porous Silica: Macromorphologies

**Figure 7:**
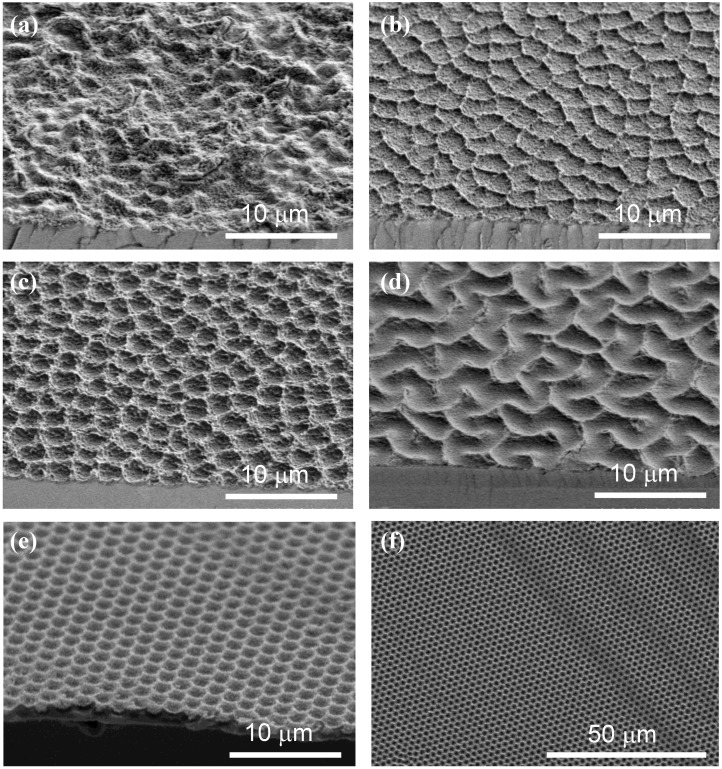
SEM views of the four non-uniform morphologies, taken at 45° near a cleavage, seen at the bottom of each image. (**a**) Disordered (0.25 M, 20 V, 20 rpm); (**b**) Waves (0.25 M, 16 V, 1,600 rpm); (**c**) Bowls (0.25 M, 20 V, 1,600 rpm); (**d**) Labyrinths (0.25 M, 45 V, 1,600 rpm); (**e**–**f**) Ordered Bowls structure obtained after prepatterning with KOH-made etch pits [(f) is a top view: note the perfect long-range ordering]. Adapted from Reference [52].

In the above, the porous oxide has been assumed to be planar and of uniform thickness. Films formed at moderate potential exhibit indeed a bright colored aspect, indicative of a planar surface and a homogeneous structure on the scale of light wavelength. However, films obtained at potentials higher than 15 V, or under low stirring conditions (low electrode rotation rate) appear dull, which suggests the presence of inhomogeneities on the micrometer scale. The structure of the porous films has been investigated systematically as a function of fluoride concentration *c*_F_, formation potential *V*, and electrode rotation rate ω, using imaging with optical microscope and scanning electron microscope [52]. Interestingly, the macrostructures appear superimposed on the mesoporosity; namely, the macroobjects observed are made of the same mesoporous material as that of the uniform films. Besides the uniform (U) morphology, four different morphologies have been distinguished (Figure 7):

- *Disordered* (D) (Figure 7a). At low electrode rotation rates and low fluoride concentrations, the top views exhibit a disordered aspect, with bright spots of a few microns in size, with random positions and distributed brightness. The roughness of the films, more quantitatively observed on cleavage planes, has essentially random character.
- *Waves* (W) (Figure 7b). In a narrow region of potential above the upper boundary of uniform film formation, the films appear decorated by protruding crests of irregular shape. SEM profiles show that the uniform pieces between the crests are slightly bent upwards. This morphology appears as a transition between the uniform morphology and the “bowl” morphology described hereafter.
- *Bowls* (B) (Figure 7c). For potentials slightly higher than those leading to the wave morphology, the top views exhibit bright circular spots, of similar size and brightness, with a tendency to short range ordering. It has been shown that prepatterning allows one to grow similar structures ordered on the long-range [27] (Figure 7e–f). The SEM profiles indicate that these spots correspond to closely circular depressions (bowls) in the surface of the film, which may look like macropores in the most extreme cases. The Si/SiO_2_ interface also exhibits bowl shapes.
- *Labyrinths* (L) (Figure 7d). For still higher potentials, the bowls tend to coalesce and to form meandering stripes. The collection of parallel stripes then exhibits a labyrinth shape.
- Finally, a fifth morphology (B’) appears at very high potentials, which has been regarded as a variant of B. However, it has a more random character and must probably be regarded as a distinct morphology, intermediate between B and D.

**Figure 8:**
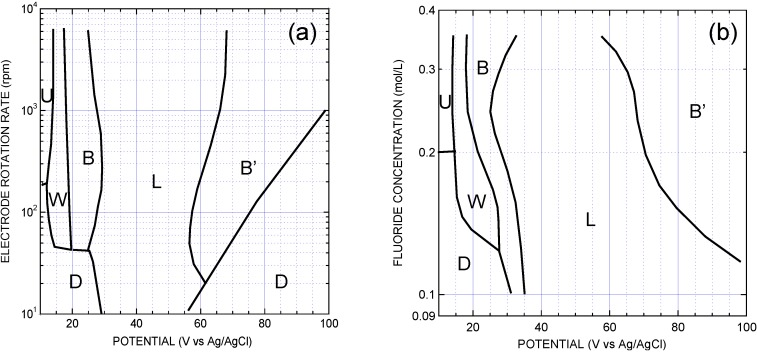
Map of the various morphologies (**a**) for an electrolyte with *c*_F_ = 0.25 M, as a function of potential and electrode rotation rate; (**b**) for ω = 1,600 rpm, as a function of potential and fluoride concentration. Uniform (U), disordered (D), waves (W), bowls (B) and labyrinths (L). B’ represents a bowls morphology with increased disorder as compared to B. Adapted from [52].

The images have been analyzed quantitatively, allowing us to define criteria to determine the boundaries between the different morphologies and to draw a map of these morphologies in 2D sections of the (*V*, *c*_F_, ω) space (Figure 8) [52]. One conclusion is that the disordered morphology, favored by low fluoride concentrations or low stirring conditions, is associated with thicker porous films. Another important conclusion is that, if the disordered morphology is avoided by using appropriate values for *c*_F_ and ω, increasing the potential leads to a sequence U → W → B → L → B’. Since increasing the potential leads to increased oxygen evolution, and especially since the onset of oxygen evolution coincides with the appearance of the non-uniform morphologies, it has been suggested that these macromorphologies are due to uneven dissolution of the porous oxide, due to local acidification of the medium by oxygen evolution. This idea is substantiated by the observation of a decrease of the average porous-layer thickness [52], in agreement with the trend predicted by the simulation shown in Figure 6. An uneven dissolution is not really surprising: if the oxide is thinner in some places, it is plausible that oxygen evolution will be favored there, leading locally to increased acidification of the medium and further favoring oxide dissolution. It is noticeable that the characteristic scale of the macromorphologies is of the same order of magnitude as the porous-layer thickness. However, a full quantitative account of the shape of the observed macrostructures would require 3D simulations of the electrochemical process, which has not been attempted at the present stage.

## 6. Porous Silica on the Nanometric Scale: The Stratified Structure

### 6.1. Observation of the Stratified Structure

Examination of the uniform morphology on a smaller scale has revealed a very surprising structure. The porous films appear formed by a stack of very thin layers of nanometric thickness. This structure is not easily observed, because silica is a very insulating material and SEM can hardly reach its optimum resolution capability. However, compelling evidence of this stratified structure has been obtained by two methods:

Grazing-incidence X-ray reflectance diagrams of films formed on a flat silicon surface exhibit a distinct peak at an angle around 0.5° and sometimes a second weaker peak at around 1°, indicating the presence of a periodic structure with a period on the order of 10 nm [53]. These data can be simulated by assuming that the modulation is a modulation of the porosity *P*, with a contrast Δ*P*/*P* on the order of a few percent [53].

**Figure 9:**
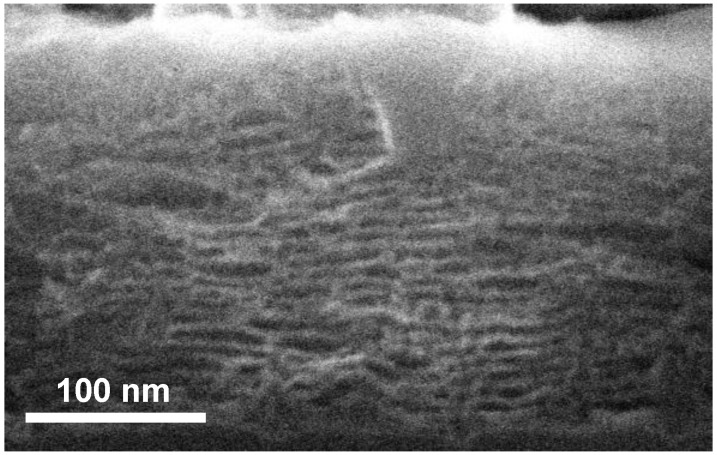
Evidence for the stratified structure of an oxide layer of uniform morphology, obtained after impregnation with electrodeposited nickel (Adapted from [54]).

Porous films have been impregnated with nickel by using electrodeposition under illumination (a requirement for a cathodic process at a p-type electrode). The deposit appears to impregnate the porous structure at some spots, and develops as spheroidal particles when it reaches the outer surface of the porous film [54]. In the regions where the nickel deposit is not too dense (near the edge of the impregnated areas), the porous structure is not appreciably disturbed, and the presence of metal allows for better SEM imaging conditions, leading to a clear-cut observation of the stratified structure on side views (Figure 9).

A study of the stratified structure over a wide range of preparation conditions has shown that it is present not only for the layers of uniform morphology, but it survives in the regime where macromorphologies are present, the direction of the layers following that of the silicon/oxide interface.

### 6.2. Origin of the Stratified Structure

**Figure 10:**
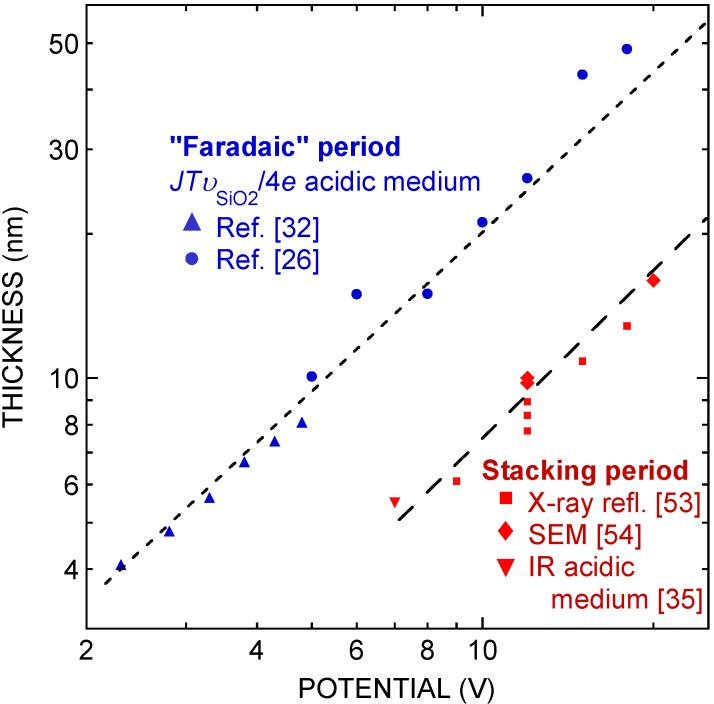
Plot of the stacking periods obtained from different experiments (see text). Note the similar order of magnitude and identical variation as a function of potential for the period observed in the oxide formed in neutral medium and for the period associated with the oscillatory behavior in acidic medium. Adapted from [54].

The magnitude of the porosity modulation period, on the order of 10 nm, is reminiscent of the oxide thickness present in the region of the second electropolishing plateau (*J*_4_ region). This suggests that the periodic structure of the layer may be associated with the periodic variation of the current associated with the oscillatory behavior observed in this regime. In order to assess this idea, we have compared the results of several experiments, by representing various characteristic lengths on a common scale, as a function of potential (Figure 10):

- The period of the porous oxide modulation, as determined by X-ray reflectivity (in the region of uniform morphology) and SEM [53,54].
- The stacking period in acidic medium, obtained from the oxide profile as a function of time. This profile was deduced from a quantitative analysis of infrared absorption data during oxide dissolution triggered at various times of the oscillation period, giving evidence for successive layers of a slowly-dissolving oxide and a fast-dissolving one [35].
- The “Faradaic period” in acidic medium, as determined by measuring the oscillation period *T* and the average current density $\bar{J}$, and calculating $\bar{J}$ *Tυ*_SiO2_/4*e*, where *υ*_SiO2_ is the volume occupied by an elementary SiO_2_ unit in silica [26,32].

These three quantities are seen to exhibit a very similar variation as a function of potential, and to be comparable in absolute magnitude. The Faradaic thickness appears to be a factor of 2–3 higher than the other two quantities. This is not surprising, because it represents the amount of silica generated during an oscillation cycle, which is expected to exceed the stacking period, since part of this silica is actually dissolved. All in all, these data provide rather convincing evidence that the stratified structure of the porous oxide is related indeed to the oscillatory behavior observed in acidic medium in the *J*_4_ region.

A layered structure of the anodic oxide has been mentioned in previous literature [55,56,57]. However, the fact that up to 50 periods can be formed and preserved upon drying the silicon electrode is rather new, and may lead to reconsidering the mechanism responsible for the oscillatory behavior. Though the very origin of the oscillatory mechanism is controversial, a common feature of all of the proposed mechanisms is the fact that the current leads to oxide growth, tending to passivate the surface, and at some point there is a breakdown of this oxide, leading to growth of a new oxide layer. The present data indicate that dissolution of the damaged oxide is not necessary, and preservation of the damaged oxide does not hinder regrowth of a new layer, even after many oscillation cycles. This can be understood if the damaged oxide is heavily pitted [33] or cracked, leading to easy ion conduction through these defects. This has led us to propose a new mechanism, termed ESC mechanism (Expansion, Shrinking, Cracking) [53]. In short, the idea is that the anodic current first leads to formation of a heavily hydrated oxide, accompanied by an expansion accommodated by the plasticity of the hydrated oxide. When this layer thickens, further oxidation leads to consumption of water molecules and hydroxyl groups, hence to dehydration of the layer. The resulting shrinking and embrittlement of the oxide lead to cracking. The electrolyte then easily penetrates through the cracks and the process can start over again. A somewhat similar mechanism, on a much longer time scale, has been invoked for the high-temperature anodization of zirconium and Zr–Sn alloy [58]. Up to now, there is no clear evidence for the presence of cracks in the porous oxide structure. However, it is our belief that it contains, engraved in its structure, useful information on the oscillatory mechanism, and by designing appropriate experiments, it should be possible to learn more about it, and clarify the mechanism responsible for this oscillatory behavior.

### 6.3. Why a Stratified Structure rather than Nanopores or Nanotubes?

**Figure 11:**
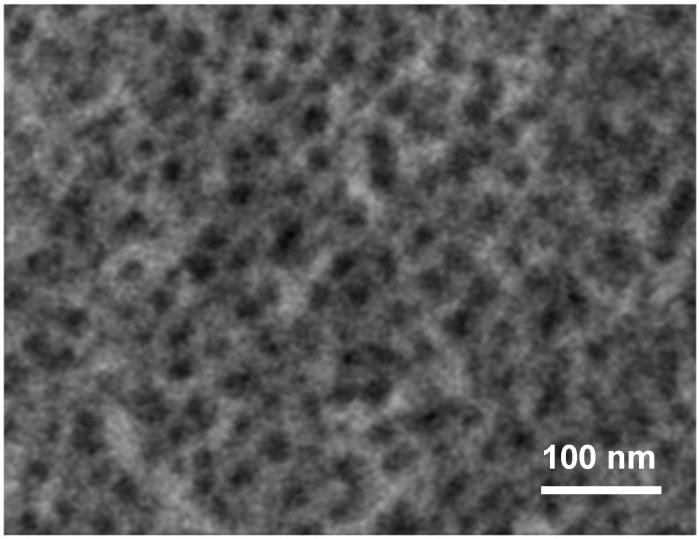
SEM front view of a porous silica layer obtained in 0.5 M NH_4_F + 0.01 M NH_4_OH solution in 90% ethylene glycol. Potential 35 V, RDE 300 rpm.

The morphologies of the porous oxide films obtained on silicon appear original as compared to the case of other materials. Aluminium leads to a nanoporous oxide, with parallel nanopores with a diameter in the range 30–100 nm [4,5]. Titanium leads to parallel nanotubes [6,7], and other valve metals (Hf, Zr, Ta, Nb, W) and alloys lead either to nanopores or nanotubes [8,9,10,11,12]. The stratified structure of the oxide observed in the case of silicon, and associated with the oscillatory behavior, seems to hinder the formation of these more common structures. As a matter of fact, the bowls macrostructure sounds a bit like aborted macropores. Interestingly, Schmuki *et al.* have recently reported that anodization of silicon in glycol-based fluoride electrolytes may lead to the formation of nanotubes [59]. However, the images are as yet very far from the nice structures obtained, e.g., on titanium. In similar conditions, our own data indicate the formation of nanopores rather than nanotubes (Figure 11). A problem is that hexafluorosilicates are sparingly soluble in glycol, and in these experiments no steady state is obtained, that is, these conditions are similar to those used in non-fluoride electrolytes. A possible explanation for the different morphologies may be that the silica formed in these conditions contains some amount of –O–CH_2_–CH_2_–O– groups substituting –O–Si–O– groups. This would lead to a softening of the material and prevent its cracking, thereby inhibiting the oscillatory mechanism. More extensive studies in non-aqueous media are clearly required to assess this hypothesis.

## 7. Conclusion and Perspectives

Electrochemistry appears to provide an original route to the preparation of porous silica on silicon substrate. The use of neutral fluoride electrolytes offers experimental conditions leading to very reproducible results, and a rich variety of morphologies may be obtained. The observation of a stratified structure of the porous films raises fundamental issues about the distinct behavior of anodic silica as compared to other anodic oxides. Its relation to the oscillatory behavior of silicon in fluoride electrolytes may provide new hints to understand this puzzling behavior. It is not clear at present whether electrochemically prepared porous silica may be of interest for applications as compared to porous silica prepared by other methods. However, we do believe that the chemical harmlessness of silica, together with the possibility to prepare it in thin-film form on a silicon substrate, should make it an interesting material for applications in the field of biomedical and bioelectronics applications.

## References

[1] Keller F., Hunter M.S., Robinson D.L. (1953). Structural features of oxide coatings on aluminum. J. Electrochem. Soc..

[2] Diggle J.W., Downie T.C., Goulding C.W. (1969). Anodic oxide films on aluminum. Chem. Rev..

[3] Thompson G.E. (1997). Porous anodic alumina: fabrication, characterization and applications. Thin Solid Films.

[4] Masuda H., Fukuda K. (1995). Ordered metal nanohole arrays made by a two-step replication of honeycomb structures of anodic alumina. Science.

[5] Jessensky O., Müller F., Gösele U. (1998). Self-organized formation of hexagonal pore structures in anodic alumina. J. Electrochem. Soc..

[6] Beranek R., Hildebrand H., Schmuki P. (2003). Self-organized porous titanium oxide prepared in H_2_SO_4_/HF electrolytes. Electrochem. Solid State Lett..

[7] Macak J.M., Schmuki P. (2006). Anodic growth of self-organized anodic TiO_2_ nanotubes in viscous electrolytes. Electrochim. Acta.

[8] Tsuchiya H., Schmuki P. (2005). Self-organized high aspect ratio porous hafnium oxide prepared by electrochemical anodization. Electrochem. Commun..

[9] Lee W.-J., Smyrl W.H. (2005). Zirconium oxide nanotubes synthesized via direct electrochemical anodization. Electrochem. Solid State Lett..

[10] Sieber I.V., Schmuki P. (2005). Porous tantalum oxide prepared by electrochemical anodic oxidation. J. Electrochem. Soc..

[11] Choi J., Lim J.H., Lee S.C., Chang J.H., Kim K.J., Cho M.A. (2006). Porous niobium oxide films prepared by anodization in HF/H_3_PO_4_. Electrochim. Acta.

[12] Yang M., Shrestha N.K., Schmuki P. (2009). Thick porous tungsten trioxide films by anodization of tungsten in fluoride containing phosphoric acid electrolyte. Electrochem. Commun..

[13] Miyata H., Suzuki T., Fukuoka A., Sawada T., Watanabe M., Noma T., Takada K., Mukaide T., Kuroda K. (2004). Silica films with a single-crystalline mesoporous structure. Nature Mater..

[14] Revesz A.G. (1967). Anodic oxidation of silicon in KNO_3_–N–methylacetamide solution: Interface properties. J. Electrochem. Soc..

[15] Gaspard F., Halimaoui A., Sarrabayrouse G. (1987). Electrical properties of thin anodic silicon dioxide layers grown in pure water. Rev. Phys. Appl..

[16] Canham L.T. (1997). Properties of Porous Silicon.

[17] Memming R., Schwandt G. (1966). Anodic dissolution of silicon in hydrofluoric acid solutions. Surf. Sci..

[18] Memming R., Schwandt G. (1966). Potential distribution and formation of surface states at the silicon-electrolyte interface. Surf. Sci..

[19] Gerischer H., Lübke M. (1988). Electrolytic growth and dissolution of oxide layers on silicon in aqueous solutions of fluorides. Ber. Bunsenges. Phys. Chem..

[20] Gershinskii A.E., Mironova L.V. (1989). Behavior of silicon in aqueous HF solutions. Soviet Electrochem..

[21] Eddowes M.J. (1990). Anodic dissolution of p- and n-type silicon: Kinetic study of the chemical mechanism. J. Electroanal. Chem..

[22] Chazalviel J.-N., Etman M., Ozanam F. (1991). A voltammetric study of the anodic dissolution of p-Si in fluoride electrolytes. J. Electroanal. Chem..

[23] Zhang X.G. (2001). Electrochemistry of Silicon and Its Oxide.

[24] Lehmann V. (2002). Electrochemistry of Silicon.

[25] da Fonseca C., Ozanam F., Chazalviel J.-N. (1996). *In-situ* infrared characterisation of the interfacial oxide during the anodic dissolution of a silicon electrode in a fluoride electrolyte. Surf. Sci..

[26] Lharch M., Chazalviel J.-N., Ozanam F., Aggour M., Wehrspohn R.B. (2003). *In situ* investigation of porous anodic films of silica. Phys. Status Solidi (a).

[27] Frey S., Grésillon B., Ozanam F., Chazalviel J.-N., Carstensen J., Föll H., Wehrspohn R.B. (2005). Self-organized macrostructures in anodically formed mesoporous silica. Electrochem. Solid State Lett..

[28] Venkateswara-Rao A., Ozanam F., Chazalviel J.-N. (1991). *In-situ* Fourier-transform electromodulated infrared study of porous silicon formation: Evidence for solvent effects on the vibrational linewidths. J. Electrochem. Soc..

[29] Belaïdi A., Safi M., Ozanam F., Chazalviel J.-N., Gorochov O. (1999). Surface chemistry during porous silicon formation in dilute fluoride electrolytes. J. Electrochem. Soc..

[30] Föll H. (1991). Properties of silicon-electrolyte junctions and their application to silicon characterization. Appl. Phys. A.

[31] Chazalviel J.-N., Ozanam F., Etman M., Paolucci F., Peter L.M., Stumper J. (1992). The p-Si/fluoride interface in the anodic region: Damped and/or sustained oscillations. J. Electroanal. Chem..

[32] Ozanam F., Chazalviel J.-N. (1992). Resonant and Nonresonant behavior of the anodic dissolution of silicon in fluoride media: An impedance study. J. Electrochem. Soc..

[33] Lewerenz H.J., Aggour M. (1993). On the origin of photocurrent oscillation at Si electrodes. J. Electroanal. Chem..

[34] Aggour M., Giersig M., Lewerenz H.J. (1995). Interface condition of n-Si(111) during photocurrent oscillations in NH_4_F solutions. J. Electroanal. Chem..

[35] Chazalviel J.-N., da Fonseca C., Ozanam F. (1998). *In-situ* infrared study of the oscillating anodic dissolution of silicon in fluoride electrolytes. J. Electrochem. Soc..

[36] Carstensen J., Prange R., Föll H. (1999). A model for current-voltage oscillations at the silicon electrode and comparison with experimental results. J. Electrochem. Soc..

[37] Grzanna J., Jungblut H., Lewerenz H.J. (2000). A model for electrochemical oscillations at the Si/electrolyte contact: Part I. Theoretical development. J. Electroanal. Chem..

[38] Grzanna J., Jungblut H., Lewerenz H.J. (2000). A model for electrochemical oscillations at the Si/electrolyte contact: Part II. Simulations and experimental results. J. Electroanal. Chem..

[39] Foca E., Carstensen J., Föll H. (2007). Modelling electrochemical current and potential oscillations at the Si electrode. J. Electroanal. Chem..

[40] Grzanna J., Jungblut H., Lewerenz H.J. (2007). Nano- and macropores in the model for current oscillations at the Si/electrolyte contact. Phys. Status Solidi (a).

[41] Hassan H.H., Sculfort J.L., Etman M., Ozanam F., Chazalviel J.-N. (1995). Kinetic and diffusional limitations to the anodic dissolution of p-Si in fluoride media. J. Electroanal. Chem..

[42] Cattarin S., Frateur I., Musiani M., Tribollet B. (2000). Electrodissolution of p-Si in acidic fluoride media-modeling of the steady state. J. Electrochem. Soc..

[43] Lharch M., Aggour M., Chazalviel J.-N., Ozanam F. (2002). Anodic dissolution and electroluminescence of p-Si at high potentials in fluoride media. J. Electrochem. Soc..

[44] Chazalviel J.-N., Ozanam F., Lharch M., Choi J., Wehrspohn R.B. Controlled formation of thick anodic films of mesoporous silica. Proceedings of 203rd ECS Meeting.

[45] Frey S., Keipert S., Chazalviel J.-N., Ozanam F., Carstensen J., Föll H. (2007). Electrochemical formation of porous silica: Toward an understanding of the mechanisms. Phys. Status Solidi (a).

[46] Chazalviel J.-N. (1992). Ionic processes through the interfacial oxide in the anodic dissolution of silicon. Electrochim. Acta.

[47] Mende G., Campbell S.A., Lewerenz H.J. (1998). Anodic oxidation of Silicon as a low-temperature passivation technique. Semiconductor Micromachining.

[48] Hoar T.P., Mott N.F. (1959). A mechanism for the formation of porous anodic oxide films on aluminium. J. Phys. Chem. Solids.

[49] Garcia-Vergara S.J., Iglesias-Rubianes L., Blanco-Pinzon C.E., Skeldon P., Thompson G.E., Campestrini P. (2006). Mechanical instability and pore generation in anodic alumina. Proc. Roy. Soc. A.

[50] Su Z.X., Zhou W.Z. (2008). Formation mechanism of porous anodic aluminium and titanium oxides. Adv. Mater..

[51] Sze S.M., Ng K.K. (2007). Physics of Semiconductor Devices.

[52] Amin M.A., Frey S., Ozanam F., Chazalviel J.-N. (2008). Macromorphologies in electrochemically formed porous silica. Electrochim. Acta.

[53] Chazalviel J.-N., Cortès R., Maroun F., Ozanam F. (2009). Stratified structure of anodically formed mesoporous silica. Phys. Status Solidi (a).

[54] Chazalviel J.-N., Ozanam F. (2010). Electrochemically formed porous silica as a template for metal electrodeposition. ECS Trans..

[55] Lehmann V. (1995). On the origin of electrochemical oscillations at silicon electrodes. J. Electrochem. Soc..

[56] Parkhutik V., Matveeva E. (1999). Observation of new oscillatory phenomena during the electrochemical anodization of silicon. Electrochem. Solid State Lett..

[57] Parkhutik V., Matveeva E., Perez R., Alamo J., Beltrán D. (2000). Mechanism of large oscillations of anodic potential during anodization of silicon in H_3_PO_4_/HF solutions. Mater. Sci. Engin. B.

[58] Schefold J., Lincot D., Ambard A., Kerrec O. (2003). The cyclic nature of corrosion of Zr and Zr–Sn in high-temperatuure water (633 K). A long-term *in situ* impedance spectroscopic study. J. Electrochem. Soc..

[59] Yang M., Shrestha N.K., Schmuki P. (2010). Toward self-ordered silica nanotubes by electrochemical anodization of Si(100). Electrochem. Solid State Lett..

